# Endocannabinoid Modulation in Neurodegenerative Diseases: In Pursuit of Certainty

**DOI:** 10.3390/biology11030440

**Published:** 2022-03-14

**Authors:** Alexandru Vasincu, Răzvan-Nicolae Rusu, Daniela-Carmen Ababei, Mădălina Larion, Walther Bild, Gabriela Dumitrița Stanciu, Carmen Solcan, Veronica Bild

**Affiliations:** 1 Department of Pharmacodynamics and Clinical Pharmacy, “Grigore T Popa” University of Medicine and Pharmacy, 16 Universitatii Street, 700115 Iasi, Romania; alexandru.vasincu@umfiasi.ro (A.V.); dana.ababei@gmail.com (D.-C.A.); veronica.bild@gmail.com (V.B.); 2 Department of Anaesthesiology Intensive Therapy, Regional Institute of Gastroenterology and Hepatology “Prof. Dr. Octavian Fodor”, 19 Croitorilor Street, 400162 Cluj-Napoca, Romania; madalina.larion@yahoo.com; 3 Department of Anaesthetics, Midland Regional Hospital, Longford Road, Mullingar, N91 NA43 Co. Westmeath, Ireland; 4 Department of Physiology, “Grigore T Popa” University of Medicine and Pharmacy, 16 Universitatii Street, 700115 Iasi, Romania; waltherbild@gmail.com; 5 Center of Biomedical Research of the Romanian Academy, 700506 Iasi, Romania; 6 Center for Advanced Research and Development in Experimental Medicine (CEMEX), “Grigore T. Popa” University of Medicine and Pharmacy, 16 Universitatii Street, 700115 Iasi, Romania; gabriela-dumitrita.s@umfiasi.ro; 7 Preclinics Department, “Ion Ionescu de la Brad” University of Life Sciences, 8 M. Sadoveanu Alley, 700489 Iasi, Romania; carmensolcan@yahoo.com

**Keywords:** endocannabinoid system, phytocannabinoids, neurodegenerative, Alzheimer’s, Parkinson’s, Huntington’s, multiple sclerosis

## Abstract

**Simple Summary:**

Neurodegenerative diseases represent an important cause of morbidity and mortality worldwide. Existing therapeutic options are limited and focus mostly on improving symptoms and reducing exacerbations. The endocannabinoid system is involved in the pathophysiology of such disorders, an idea which has been highlighted by recent scientific work. The current work focusses its attention on the importance and implications of this system and its synthetic and natural ligands in disorders such as Alzheimer’s, Parkinson’s, Huntington’s and multiple sclerosis.

**Abstract:**

Neurodegenerative diseases are an increasing cause of global morbidity and mortality. They occur in the central nervous system (CNS) and lead to functional and mental impairment due to loss of neurons. Recent evidence highlights the link between neurodegenerative and inflammatory diseases of the CNS. These are typically associated with several neurological disorders. These diseases have fundamental differences regarding their underlying physiology and clinical manifestations, although there are aspects that overlap. The endocannabinoid system (ECS) is comprised of receptors (type-1 (CB1R) and type-2 (CB2R) cannabinoid-receptors, as well as transient receptor potential vanilloid 1 (TRPV1)), endogenous ligands and enzymes that synthesize and degrade endocannabinoids (ECBs). Recent studies revealed the involvement of the ECS in different pathological aspects of these neurodegenerative disorders. The present review will explore the roles of cannabinoid receptors (CBRs) and pharmacological agents that modulate CBRs or ECS activity with reference to Alzheimer’s Disease (AD), Parkinson’s Disease (PD), Huntington’s Disease (HD) and multiple sclerosis (MS).

## 1. Introduction

Neurodegenerative diseases are an increasing cause of global morbidity and mortality [1]. Pressure exerted on healthcare systems by diseases such as PD or AD make them a priority for healthcare workers [2].

It was found that in the USA, between 2016 and 2017 there were approximately 4.7 to 6.0 million individuals living with neurodegenerative disorders, which were responsible for 272,644 deaths [3]. In 2014, 1.6% of the US population lived with AD and related dementias. In 2020, the cost for AD, amyotrophic lateral sclerosis (ALS), PD and spinal muscular atrophy was $655 billion [4,5]. It is estimated that by 2060, the prevalence will double to 3.3%, meaning that approximately 13.9 million adults will be living with AD and related dementias [6]. Regarding the EU, studies show that in almost twenty years (1994–2013), deaths attributed to AD increased from 41,255 to 86,822 [7]. These numbers highlight the significant impact of these disorders on society and patients.

The neurodegenerative processes behind these disorders occur in the CNS and are associated with the alteration of neuronal morphology and functionality, thus leading to functional and mental impairment due to reduced neuronal survival and increased neuronal death [8,9].

Recent evidence also highlights the link between neurodegenerative diseases and inflammatory diseases of the CNS. Neuroinflammation is a protective mechanism of the CNS due to the activation and proliferation of B- and T-cells, neutrophils, macrophages, microglia and mast cells within the spinal cord and brain, with removal or inactivation of noxious agents such as viruses, bacterial lipopolysaccharides and inhibition of different pathogens. The persistence of the neuroinflammatory state activates microglia and astrocytes [10]. Axonal degeneration, synaptic impairment, mitochondria dysfunction, degradation of proteins and apoptosis are due to the release of various neurotoxic cytokines and chemokines [11].

These conditions are recognized in different neurological disorders, including AD, PD, HD and MS [12]. While MS is a classical example of a neuroinflammatory disease with aspects of neurodegeneration, the other three disorders have as a common feature the aggregation of misfolded proteins [13]. The degenerative disorders are demonstrably affected by changes in their conformation, thereby gaining toxicity or losing physiological functions [14], and by inflammation [15]. These diseases are the most commonly occurring forms and have fundamental differences regarding their underlying physiology and clinical manifestations, although there are aspects that overlap, such as changes in the morphology of microglia, progressive neuronal loss, oxidative stress and elevated cytokine levels [16].

This complex cascade of underlying mechanisms shows that neurodegeneration as well as neuroinflammation play important roles in these types of disorders. By tackling these two components, novel therapeutic strategies could be developed.

*Cannabis sativa* (family *Cannabaceae*), commonly called marijuana, has been widely used throughout history for medical and therapeutic purposes. There is evidence that its use dates back to 5000 years ago in what is today known as Romania [17].

The waxy resin obtained from leaves and flowers of female plants contains approximately 100 lipid soluble compounds with pharmacologically relevant activity (“cannabinoids” or “phytocannabinoids”) [18,19]. Two of these compounds are of major significance: cannabidiol (CBD) and Δ^9^-tetrahydrocannabinol (Δ^9^-THC). The structure of Δ^9^-THC, which is the main psychoactive phytocannabinoid, was determined in 1964 by Mechoulam and Gaoni [20,21]. Results of their work determined further research and, in the early 1990s, specific membrane receptors of Δ^9^-THC were discovered and opened the way to the revelation of a novel receptor system, a system that is known to us today as the ECS [22].

Δ^9^-THC and CBD are two extensively studied phytocannabinoids with positive effects in different neurodegenerative diseases [23]. The main difference between Δ^9^-THC and CBD is the fact Δ^9^-THC induces psychotropic effects while CBD is non-psychoactive and could reduce the side-effects of Δ^9^-THC [24].

Recent studies have highlighted the importance of the ECS in regard to mechanisms that underlie neurodegenerative diseases [1]. The complexity of ECS offers new perspectives into the mechanisms of neurodegenerative and neuroinflammatory components of diseases such as AD, PD, HD and MS, contributing to a more profound view on the pathophysiological aspects and thus allowing for the discovery of potential targets for their treatment [15].

The present review will explore the roles of CB1R and CB2R, and pharmacological agents that modulate CBRs or ECS activity, with reference to AD, PD, HD and MS diseases.

## 2. The Endocanabinoid System

ECS is a ubiquitous and complex component that influences numerous parts of the physiology and pathology of mammals [1].

This system influences a number of cognitive and neurophysiological processes such as motor function, memory, learning, energy metabolism, inflammation, nociception and neuroprotection, making it an attractive pharmacological target [25,26].

ECS consists of receptors, enzymes and transporters that regulate ECB-actions at the central level, as well as in the periphery. Important receptors are those that bind extracellularly, which are type 1 and type 2 receptors (e.g., CB1R and CB2R), ones that bind intracellularly (e.g., TRPV1), as well as receptors that are coupled to G-protein [27]. Regarding endogenous ligands, anadamide (AEA) and 2-arachidonoylglycerol (2-AG) are of importance, while the main enzymes that contribute to the metabolism of ECBs are N-acylphosphatidylethanolamine phospholipase D (NAPE-PLD), monoacylglycerol lipase (MAGL), and fatty acid amide hydrolase (FAAH) [28,29,30,31].

### 2.1. Endocannabinoids

Endocannabinoids (ECBs) are messengers of endogenous lipids, their name being attributed to their property to bind to CBRs.

As mentioned above, the most widely studied ECBs are AEA and 2-AG, which are derived from arachidonic acid [25]. These are involved in retrograde synaptic transmission by inhibiting the release of neurotransmitters at presynaptic level [32]. Other ECBs are virodhamine, noladin ether and *N*-arachidonoyldopamine, besides homo-linolenylethanolamide and docosatetraenylethanolamide [33].

The first endogenous compound identified was AEA but studies have shown that 2-AG would be a more appropriate endogenous ligand of CBRs, being the most abundant ECB in the brain [25,34]. They act as neurotransmitters being synthesized and released by neurons but differ from classical neurotransmitters in that they are synthesized and released when required, without being stored in synaptic vesicles, in a retrograde manner from postsynaptic neurons, and subsequently act on presynaptic CBRs [35,36,37].

The mechanism of action of ECBs is unconventional because it consists in synaptic retrograde transmission. It involves second messenger pathways such as cyclic AMP-increased intracellular Ca^2+^ concentrations that activates different protein kinases using as activation molecules the cannabinoid ligands, with particular importance placed on the control of multiple metabolic pathways carrying endogenous cannabinoids [38,39].

By gating postsynaptic-located glutamate receptors, glutamate is released under the flow of calcium ions into the synaptic cleft. The release of glutamate from the synaptic cleft into the presynaptic space is due to excessive presynaptic activity [25,37]. The process involves the activation of presynaptic metabotropic receptors triggering enzymatic activity with production of 2-AG. The ECB, released postsynaptically, moves retrogradely through the synaptic cleft, has intrinsic activity and activates CB1R and CB2R presynaptically, whereas AEA has low affinity for CB1R and very low affinity for CB2R [38]. This process involves second messenger pathways such as cyclic AMP-increased intracellular Ca^2+^ concentrations that activate different protein kinases [40,41].

Generally, ECBs act as retrograde synaptic signals by stimulating type 1 receptors causing suppression of neurotransmitter release. Regardless of the presynaptically released neurotransmitter (gamma-aminobutyric acid (GABA), glutamate) or the endogenous or exogenous cannabinoid ligand activating CB1R, the stimulation will always lead to decreased presynaptic transmission via retrograde control [42].

ECBs exert their neuroprotective roles while released in response to pathological factors. AEA and 2-AG have been found in the ventral tegmental area (VTA), where they act as retrograde messengers by activating CB1 receptors located on excitatory as well as inhibitory synapses. The main neurons in the VTA contain dopamine and play a major role in reward, cognition and motivational behaviors [26].

### 2.2. Endocanabinoid Receptors

Among numerous receptors of ECBs ligands described in the literature, the most studied are mainly CB1R, encoded by CNR1 gene and CB2R, encoded by CNR2 gene [43,44]. Through adenylyl cyclases inhibition, voltage-gated calcium channels, inward rectifying potassium channels and the activation of mitogen-activated protein kinases (MAPK), CB1R and CB2R signal transduction is mediated [45,46].

It has been suggested that cannabinoid ligands can bind and activate other targets such as GPR 18, GPR 55, GPR 119, TRPV1 and TRPV2 receptors [47].

The most abundant receptor is CB1R, which is mainly expressed centrally in neurons and glial cells. Neurological functions (memory, cognition) and emotional and pain control are modulated by the activation of this type of receptor [48,49,50].

Through proteomic system analysis and information from different sources on the mRNA, it has been shown that CB1R can be found at the mRNA level in the brain, in the pituitary gland, in adipocytes, and at the protein level in the central nervous system. CB2R can be found in blood and at the mRNA level in lymphoid tissue while also being present in different protein levels in the majority of tissues [39].

Likewise, it was shown that CB1R was mainly present in the plasma membrane and in the actin filaments, while CB2R was also found in the plasma membrane as well as in B- and T- type lymphocytes and in alveolar type 2 cells. These findings were suggested by studies that used single-cell RNA sequencing information from human normal tissues as well as protein localization information obtained from antibody-based profiling in the Cell Atlas and the HPA Cell Type Atlas [51].

In terms of localization at the central level, CB1Rs are found almost in all synapses, mainly in the basal ganglia, hippocampus, cerebellum, neocortex and hypothalamus [25,52]. CB1R can be found in presynaptic or postsynaptic levels in glutamatergic synapses, being able to control excessive-glutamate release and an overactivation of glutamatergic receptors [53]. At the same time, it can also limit the influx of calcium as well as the activation of destructive-pathways, which are calcium-dependent [54]. These receptors are also present in GABA-ergic neurons, where they are silenced under excitotoxic conditions, unlike in glutamatergic neurons, where they are activated [55,56].

CB1R can also be found in the peripheral tissue such as in the liver, kidneys and lungs, where it modulates metabolism and energy balance [57,58]. Its signaling is involved in different pathologic processes of movement disorders reproduced in animal models [59].

CB2Rs were considered “peripheral cannabinoid receptors” due to the fact that they are mainly located on endocrine and immune cells and peripheral organs (e.g., muscle, intestine, liver and testicles) [53]. High CB2R mRNA was also expressed in the spleen [48,60]. Its expression can increase up to 100-fold in inflammatory processes, after injuring the tissues, in brain trauma, or in various pathological states [25,52]. CB2R can also be found in CNS within astrocytes, brainstem and microglia in stressful conditions such as neuroinflammation. It is not present in resting microglia [61,62,63]. Upon the activation of CB2R from microglial cells, the release of anti-inflammatory cytokines has been induced, while proinflammatory cytokines and their release are inhibited [64].

Cannabinoids also have affinity to another group of receptors called vanilloid or capsaicin receptors (TRPV1) that are located on peripheral nervous system. Their presence has also been observed in sensory neurons in the skin, heart, blood vessels or lungs, and in CNS. TRPV1 receptors play an important role in transmission and modulation of pain. Their activation by AEA releases substance P and calcitonin gene-related with promotion of local vasodilation and inflammation [49,65].

### 2.3. Neuroprotection Roles

Components of ECS play a major role as key regulators of the immune system within neurodegenerative diseases, with important immunomodulatory effects in the brain, promoting neuroprotection by decrease of neuroinflammatory process and of its important triggers, such as oxidative stress, cellular apoptosis or toxic neural excitability.

The reduction in the immune response is mostly due to CB2R activation in microglia and brain-infiltrating immune cells. However, recent studies also demonstrate that CB1R helps regulate the immune system in the case of MS or AD models or traumatic brain injury [66,67,68,69].

Increased levels of ECBs (AEA and 2-AG) in the brain through inhibition of FAAH and MAGL enzymes are a successful therapeutic option for controlling the immune response in AD, HD and MS disorders [12,68].

Several cellular mechanisms such as inhibition of both glutamate release at presynaptic level and NMDA-induced intracellular Ca^2+^ release, antioxidant activity, protein kinase A (PKA) signaling and nitric oxide generation contribute to the neuroprotective effect of type 1 CBR agonists on in vitro excitotoxicity, which is induced by *N*-methyl-D-aspartic acid (NMDA). Thus, neuroprotective properties of cannabinoids suggest their potential therapeutic use in managing neurodegenerative disorders (e.g., multiple sclerosis) [53].

Nowadays, the anti-inflammatory and anti-nociceptive roles of ECBs are widely documented [70].

ECB molecules modulate nociception through central excitability and control of transmission of pain information to the CNS. The relationship between elevated concentrations of ECBs and pain is not fully established due to its involvement in both analgesic and pro-algesic phenomena. The analgesic effects of ECBs are due to CBRs activation, while the pro-algesic effects are exerted by production of arachidonic acid and of its active metabolites such as prostaglandin E2 (PGE2). Cyclooxygenase (COX)-2 pathway of ECBs metabolism could promote these antagonistic effects, depending on the metabolite produced [71].

Additionally, their role in neuroprotection has been shown by their involvement in modulation of the Blood–Brain Barrier (BBB) and further control of pathogens and toxic compounds, which circulate the blood at the brain level. In vitro/in vivo models of multiple sclerosis, chronic head injury and ischaemia/reperfusion have revealed that AEA and 2-AG have a regulating effect on its permeability [72].

This is of importance because the main pathological features of neurological diseases such as chronic inflammatory demyelinating polyneuropathy, AD, MS and neuromyelitis optica are different dysregulations of the BBB. In addition to the neuroprotective effects mediated by CB1R and CB2R (e.g., decrease of inflammatory state and reduction of cellular death as well as oxidative stress), it was also shown that they are capable of restoring and also improving the integrity of the BBB, contributing to the protective effects in psychiatric and neurological diseases [73].

Another way through which these receptors exert their protective effect on the BBB is through clearing it from Aβ depositions by contributing to their efflux. Because of a dysregulation in the BBB, Aβ cannot be transported across the barrier (Aβ-depositions are an important feature in AD). The involvement of the ECS in the transport of Aβ across the BBB was highlighted by Bachmeier et al. in a study that used animal models of AD in which depositions of Aβ were reduced in the AD brain, while cognitive behavior was improved [30].

As mentioned beforehand, the most common neurodegenerative disorders are AD and PD, age-dependent conditions characterized by memory and cognitive impairments (in AD) or affected ability to move, speak and breathe (in PD). Both diseases have common pathophysiological factors, including neuroinflammation and oxidative stress [74,75].

Due to their ability to modulate a wide range of cognitive functions (e.g. memory, learning, affective parameters, etc.) via their interactions with neurotransmitters, cannabinoids could have a positive effect on these type of diseases [52,76].

## 3. Cannabinoids and Alzheimer’s Disease

AD is one of the most serious CNS diseases, characterized by neuronal degeneration with irreversible evolution, and clinically it is manifested by impairment of cognitive functions often accompanied by other disorders such as behavioral, verbal, motor, mood and functional disorders, altering patients’ quality of life [75,77,78]. Major brain lesions are caused by the formation of extracellular senile plaques. Their structure includes insoluble β-amyloid (Aβ) peptides that initiate a neurodegenerative cascade resulting in neuronal cell death. Another specific feature of AD is intracellular tau proteins associated with microtubules, folded and hyperphosphorylated, which clump and form neurofibrillary tangles (NTFs), disrupting nutrient transport and causing neuronal senescence [35,75,79,80]. Based on these aspects, cannabinoids have been found to have neuroprotective effects.

Some preclinical studies suggest that CB1R activation causes hyperpolarization of the neuronal membrane, modulating neurotransmitter and cytokine release. Increased CB1R density in AD may be considered as a neuroprotective and anti-inflammatory response that compensates for excitotoxicity, with these findings suggesting that CB1R agonists could promote neuronal survival and could also be considered as therapeutic targets for treating agitation in AD [79].

Learning and memory impairment can be considered the most relevant side effects of cannabinoids, effects mainly mediated by CB1R through their activation. These issues have been observed in numerous studies conducted on rats and mice, which have evaluated the effects of agonists such as arachidonyl-cyclopropyl amide (ACPA) and antagonists (AM251) of CB1Rs, on spatial memory and learning processes, where agonists caused cognitive impairments and antagonists often enhanced memory and learning processes [52,81].

Low serotonin synthesis is involved in determining the occurrence of mood and behavioral disorders, as CBD and THC may contribute to increased synthesis of tryptophan, a precursor for serotonin synthesis. Compensating for the degradation of tryptophan could be an important mechanism by which CBD and THC can improve status in diseases associated with inflammation because it passes through one of the metabolism pathways into serotonin, a neurotransmitter involved in the pathogenesis of mood disorders [82].

Cannabinoids are involved in the reduction of oxidative stress and of the tumor necrosis factor-α (TNF-α); they may have synergistic effect on acetylcholinesterase (AchE) inhibitors, preventing Aβ formation, and they may improve circadian rhythm disturbances commonly seen in AD [79,83].

The proteolytic cleavage of APP generates Aβ1-42 monomer, which, under pathological conditions, leads to the formation of oligomers that activate astrocytes and microglia, release kinases, release nitric oxide, produce inflammatory cytokines and promote the phosphorylation of tau proteins.

Microglia and astrocytes in the presence of inflammatory processes synthesize ECBs (AEA and 2-AG) that suppress cytokine synthesis via CB1/CB2 receptors. In AD progression, nuclear factor-κB (NF-κB) activates the β-secretase (BACE1) promoter favoring increased synthesis of Aβ1-42 [84]. The neuronal protective role of ECBs against beta-amyloid-induced neurodegeneration has been demonstrated on primary hippocampal neuron cell cultures from rat embryos. After sacrifice, embryos were removed, from which hippocampi were collected and prepared for determination of TUNEL (neurodegenerative marker)-positive neurons. The number of TUNEL-positive neurons was significantly increased in the Aβ 25-35-treated culture, but Aβ 25-35-induced neurodegeneration and apoptosis were prevented by application of 2-AG as this protective effect appears to be mediated by CB1R. This ECB is metabolized in the brain by hydroxylation reaction in the presence of monoacylglycerol lipase (MAGL). It was observed that administration of MAGL inhibitors (URB 602 and JZL 184) increased endogenous levels of 2-AG with significant reduction of TUNEL-positive neurons in cultures treated with Aβ 25-35 or Aβ1-42. The results of this study suggest that 2-AG has anti-apoptotic effects that are concentration-dependent [85]. In another study, it was shown that this ECB is able to suppress the expression of COX-2, an enzyme involved in neuroinflammation, action also mediated by CB1R [86].

Another mechanism through which ECS confers neuroprotective effects is related to the direct clearance of Aβ via CB2-activated, macrophage-induced phagocytosis and through the inhibition of glycogen synthase kinase-3β (GSK-3β), which in turn has an attenuating effect on the hyperphosphorylation of tau protein [75].

CB2R is vital for memory as cognitive process, while a depletion or disruption of these receptors in rodents has been shown to induce long-lasting memory deficits. Administration of CB2R antagonists, such as AM630, in rats showed negative effects such as impaired memory. However, administration of CB2R agonists (JWH-133) in rats significantly reduced spatial memory impairment, memory impaired by administration of okadaic acid (OKA), which mimics AD due to tau pathology. The beneficial effects of this agonist were also manifested by reduced neuroinflammation and neurodegeneration [87].

The neuroprotective effects of CBD have been demonstrated in β-amyloid-induced AD, with the formation of Aβ peptide aggregates in the brain. In this condition, massive loss of hippocampal neurons occurs, leading to biochemical communication breakdown between neurons, subsequently followed by neuronal apoptosis [88]. CBD interacts with the PPAR-γ receptor, attenuating beta-amyloid (Aβ)-induced neuroinflammation and also promoting neurogenesis in the hippocampus [89].

In recent decades, research or studies have shown that CBD may be a potential drug for improvement of cognition within AD. A recent study attempted to discover the molecular mechanism by which CBD improves the pathology of AD.

Hippocampal transcriptome sequencing from 6-month-old APP/PS1 mice after CBD treatment (intraperitoneal (i.p.) administration, daily for one month) was used as research methods to investigate AD progression. Autophagosomes were analyzed by electron microscopy showing significant enhancement, and Aβ plaques were investigated by immunohistochemistry, these being significantly lower in the CBD treated group [90].

Another study showed the cerebroprotective action of some cannabinoids, which may be due to the mechanism of inhibition of myeloperoxidase independent of the cannabinoid receptor. The study was performed on a murine model (male mice) induced with focal cerebral ischaemia. In this study, Δ^9^-THC and CBD significantly reduced the size of cerebral infarction given by middle cerebral artery (MCA) occlusion. Δ^9^-THC interacts with central CB1Rs and peripheral CB2Rs, leading to affective, cognitive, sensory and somatic effects. It also has neuroprotective properties in cerebral ischaemia and hypoxia, improving ocular circulation [52,75]. Treatment with Δ^9^-THC before ischemia challenge reduced infarct size, while pre- and post-ischemic treatment with CBD showed strong and longer-lasting neuroprotection. CBD also inhibited myeloperoxidase activity in neutrophils, acting through an anti-inflammatory mechanism independent of cannabinoid receptors. The results of this study indicate that CBD alone has a pre- and post-ischaemic neuroprotective effect independent of cannabinoid receptors, offering the possibility of a new therapeutic perspective for neurodegenerative disorders [91].

The CBD proved its anti-inflammatory actions through its ability to decrease reactive oxygen species (ROS), thereby inhibiting inflammation not only in AD but also in murine models of type 1 diabetic cardiomyopathy, colitis, inflammatory bowel syndrome and pneumococcal meningitis [89].

The use of exogenous cannabinoids such as CBD has led to positive results in neuronal cell cultures exposed to β-amyloid. Neuroprotective properties have been demonstrated in numerous studies of mice and rats whose Aβ-induced memory deficits were prevented by exogenous cannabinoids, while CBD administration determined/promoted neurogenesis, highlighting the potential beneficial effects in AD. Other studies have shown that cannabinoids improve brain perfusion, behavioral disturbances in AD [35]. An overview of recent literature data regarding the involvement of ECS in various pathological aspects of AD is presented in Table 1.

## 4. Cannabinoids and Parkinson’s Disease

PD is a chronic neurodegenerative disease, its main feature being the loss of dopaminergic neurons in the substantia nigra of the basal ganglia. This loss is generated by various factors such as inflammation, oxidative stress, genetic mutations and misfolded protein aggregates.

In terms of clinical manifestations, the ones specific to this condition are muscle rigidity, bradykinesia, postural instability, tremor, sleep disturbances, fatigue, uncoordinated body movements, amnesia, voice disorders and cognitive dysfunction [75,101].

In patients with PD, an increase in CB2R was observed compared to controls with unchanged CB1R, with these differences suggesting the main role of CB2R in the pathogenesis of PD [101].

Endogenous and exogenous cannabinoids play a pivotal and complex role in basal ganglia circuitry through interactions with dopaminergic, glutamatergic and GABAergic mechanisms. Dopaminergic transmission at this level is modulated by the ECS and by activation of of presynaptic CB1R, and the release of GABA from striatal terminals that project to dopaminergic neurons is decreased. By activating CB1R and CB2R, an inhibition of the release of glutamate from the cortical axon head that projects to the striatum is observed. By influencing the release of neurotransmitter, dopamine synthesis in the dorsal striatum is indirectly affected, even though CB1R are not found on dopaminergic neurons [37]. In PD, motor activity is intensely affected. In order to better understand this disorder, we will review the role of the basal ganglia, which are primarily responsible for motor activity but also for executive behavioral functions [102].

Spinal projection neurons (MSN) receive signals from the glutamatergic cerebral cortex, an excitatory neurotransmitter; these neurons are modulated by nigrostriatal dopaminergic transmissions by stimulating D1 and D2 receptors followed by the generation of excitatory or inhibitory effects [37].

Presynaptic CB1R activation in the striatal area has an inhibitory effect on motor activity. CB1R are presynaptic regulators of synaptic transmission, which means that controlling excess presynaptic activity should be one of the main applications. It is well known that excitotoxicity causes lesions [25,37].

Thus, ECS disruption is involved in the neurodegenerative component of PD due to its’ impairment in the basal ganglia [101].

The neuroprotective effect of cannabinoids has been highlighted by numerous studies on animal models of PD. Experimental research has shown that three weeks after injecting mice with 1-methyl-4-phenyl-1,2,3,6-tetrahydropyridine (MPTP), CB2R have been down-regulated in the hippocampus and in the substantia nigra [103]. Thus, on the MPTP-induced mouse model, administration of JW015, a CB2R agonist, protects neuronal loss as well as MPTP-induced motor deficits. Administration of AM1241, a selective CB2R agonist, regenerated dopaminergic neurons in MPTP-induced PD mice [104].

It is also well known that chronic administration of levodopa (L-DOPA) for motor symptoms can induce dyskinesia, with the ECS being a target for attenuation of dyskinesia, which has been demonstrated in animal models [37].

Studies in experimental rodent and primate models of PD have shown profound changes in CB1R signalling in basal ganglia circuits. Other studies in animal models and in PD patients have shown that the disease is linked to an overactivation of the ECB signalling system, with increased levels of AEA and 2-AG, as well as an increase in the CB1R density, an aspect which has been shown post-mortem in the striatum of PD patients.

This increase in CB1R density can be considered as the compensatory response that occurs due to dopamine depletion in PD.

CB2Rs also play a particularly important role and are involved in neuroinflammation in PD. In post-mortem studies in PD patients, it has been shown that CB receptors in microglial cells manifested increased density [25,105].

Numerous studies in animal models of PD have shown that pharmacological activation of CB2Rs suppressed the release of pro-inflammatory cytokines, selective agonists of CB2R with the ability to reduce inflammation in the brain of mice treated with MPTP, while pharmacological inactivation of these receptors resulted in reversal of this effect. Furthermore, CB2R-deficient mice showed an exacerbation of PD pathology [25].

Currently, the treatment for PD aims for the management of symptoms, with its efficacy being limited by its side effects. It is well known that L-DOPA therapy can cause dyskinesia, which could be antagonized or prevented by the administration of cannabinoids exerting their anti-dyskinetic effect. GABA-induced signal transmission was enhanced by cannabinoid agonists through inhibition of its uptake at glutamatergic synapses and suppression of excitation produced by NMDA receptors and AMPA receptors on dopaminergic neurons, contributing to the anti-dyskinetic effect [75]. Modulation of dopaminergic activity by the ECS in the basal ganglia has been supported by various behavioral, electrophysiological and neurochemical studies [101].

The neuroprotective properties of CBD have been increasingly scientifically evaluated in neurodegenerative diseases, including PD and AD [106,107]. Although studies in animal models of PD are promising, clinical studies evaluating the neuroprotective effects of CBD are few. A study from 2014 reports that 21 PD patients selected from a sample of 119 patients received a daily dose of 300 mg CBD. Patients were assessed before the start of treatment as well as in the last week of treatment, with motor score, symptoms present in PD and change in patients’ lives for possible neuroprotective effects assessed. The results of this study suggest that CBD could improve patients’ quality of life but only in those cases free of comorbidities [106]. An overview of recent literature data regarding the implications of ECS in various pathological aspects of PD is presented in Table 2.

## 5. Cannabinoids and Huntington’s Disease

HD, also called as Huntington’s chorea [110], is a rare neurodegenerative autosomal dominant disorder, being the most common monogenic neurological disorder in the developed world [111]. The mean age at onset is 40 years, whereas recent prevalence studies of disease in population was between 5 and 10 persons per 100,000 [112]. The survival of patients with HD is about 17 years [113].

HD has autosomal dominant inheritance [114,115], being characterized by a repeated expansion of CAG trinucleotide in the first exon of the Huntingtin (HTT) gene, located on chromosome 4/band 16.3 [116]. The gene encodes HTT affected proteins, in which the expansion of CAG repeats leads to expansion of glutamine repeats [115,117]. The hallmark of the disease consists in accumulation of mutant Huntingtin (mHTT) aggregates and inclusions throughout the brain. Despite being caused by unrelated proteins with different expression patterns, other disorders such as AD, PD and ALS have a set of common characteristics with HD [112].

The symptoms and signs of HD consists of motor disturbances, behavioral and psychiatric symptoms and cognitive disturbances [118].

Numerous agents and interventions have been assessed for their efficacy in suppressing HD-chorea. These agents include antagonists and agonists of dopamine, drugs that deplete dopamine, inhibitors of acetylcholinesterase, benzodiazepines, glutamate antagonists, antiepileptic drugs, fetal cell transplantation and deep brain stimulation [119,120,121,122]. The current available options are symptomatic, without an influence on the course of the disease. Pharmacological interventions usually address the movement disorders associated with the disease [123]. The chorea-component is treated using tetrabenazine, which was approved by the FDA for this use in 2008. In 2017, FDA approved deutetrabenazine, a second therapeutic option for the treatment of chorea and for tardive dyskinesia, with improved pharmacokinetic profile. Excitotoxicity and neuronal death can be caused by an excessive increase in glutamate release; thus, treatments may involve the blockage of glutamatergic receptors or the inhibition of glutamate release. Drugs that demonstrated efficacy in clinical trials and act upon glutamatergic transmission are memantine, amantadine, lamotrigine and remacemide [124]. In the case of patients with Westphal variant, an akinetic form of the disease, drugs such as dopamine agonists, amantadine, levodopa and antiparkinsonians could be of use [123,125,126,127].

Studies using mouse-models of HD demonstrated an impairment in the function of ECS. In patients with HD, studies that have been realized on postmortem brains have shown a decrease of CB1-immunoreactivity throughout the course of the disease in the globus pallidus as well as in the putamen.

In vivo PET imaging has shown that even in early manifest phases in patients with the manifest form of the disease, a loss of CB1R binding is present [128]. This suggests that a reduction of type-1 receptors could be seen even in the pre-HD phase, hypothesis also supported by observing transgenic mouse models of the disease in the pre-HD phase, in which a reduction of levels of CB1R protein and messenger RNA was present. During the manifest course of the disease, a further decline is noticed [129]. Ceccarini et al. has also managed to demonstrate that a reduction of CB1R is present in pre-HD. This could be caused by the mHTT and its repressive effect on the transcription of the CB1R gene [130]. The reduction was considered a potential compensatory response because it could reduce GABA release in the striatum [131,132].

An early neurochemical alteration, which can be seen in HD patients, refers to a reduction of striatum-CB1R binding. It seems that CB1R mRNA decrease appears before the appearance of motor symptoms in transgenic rodent models of HD [133]. Glass et al. has shown that environmental enrichment determines an upregulation of CB1R binding in the R6/1 transgenic model of HD, thus providing behavioral improvement [134]. It is thus suggested that a protective effect could be obtained by activating CB1R, while a reduction of these receptors can negatively influence the disease. By analyzing post-mortem brains of patients suffering from HD, a decreased of CB1R expression was observed. This reduction was also seen in genetic and phenotypic models of the disease. It was shown that an up-regulation of CB1R led to an improvement of motor dysfunction, leading to the idea that an early reduction of these receptors is of high importance in HD development [135].

Work conducted by Stephane et al. on phenotypic 3NP and N171-82Q transgenic model of the disease has highlighted the involvement of CB1R through the inactivation of its specific gene: in the N171-82Q model, loss of CB1R is associated with an early appearance of motor symptoms and an exacerbation of these alterations, in a similar way to that seen in R6/2 model [133]. It also determines an increase in striatal aggregation frequency in N171-82Q model similar to the R6/2 model [136]. Regarding the excitotoxic-dependent 3NP model, it has been shown that CB1R is necessary to counteract neuronal degeneration [133].

In a study realized by Laprairie et al., it has been shown that CB1-positive allosteric modulators (PAM) (GAT211, GAT228 and GAT229) determined improvements in R6/2 mouse model of the disease through their beneficial influence on the viability of mHTT-expressing cultured medium spiny projection neurons. The CB1-selective aimed to evaluate the positive outcomes of allosteric modulation of type 1 receptor in HD. GAT211 and its enantiomer GAT229 determine CB1-dependent effects in vivo, leading to a reduction in psychoactivity without tolerance or dependence [137,138,139]. Thus, positive allosteric modulation of CB1 could reduce the progression of the disease while also delaying it [138].

Unlike neuronal CB1R, microglial CB2R are induced in HD animal models and patients. Palazuelos et al. have shown that in a transgenic model of a neurodegenerative disease, CB2R ablation determined an exacerbated activation of the microglia, contributing to the onset of symptoms [140]. In an experimental malonate-rat model of HD, an increase in CB2R could be observed in reactive microglial cells and in activated astrocytes within the striatum [141]. Unlike CB1R orthosteric agonists, which induce psychotropic effects, mood alterations, cognitive and motor impairments and acute psychosis, CB2R ligands appear to be promising drugs for treating neuro-inflammatory diseases [142] because they do not induce the undesirable psychotropic effects. Thus, CB2R activation is associated with a neuroprotective effect in HD models through controlling the damaging activity of the microglia [140].

CB2R selective agonists may represent an important therapeutic alternative to CB1R due to the early reduction of CB1R during HD and to the lack of psychotropic effects of CB2R agonists. They are viable options for the potential treatment of the disease because of their ability to reduce non-cell autonomous degeneration of neurons by active microglia [140]. The major challenge regarding CB2R is related to the selective targeting of the brain CB2Rs without influencing peripheral CB2R. It is known that CB2R levels are much higher in peripheral tissues, with this type of receptor being considered a “peripheral cannabinoid receptor”.

Drugs that are currently used in clinics and activate CB1R and CB2R are nabilone, dronabinol, Δ^9^-THC and combination of Δ^9^-THC with CBD. Considering that the side effects of these agonists are due to the activation of CB1R, it is anticipated that new drugs that selectively modulate CB2Rs will emerge [143].

A 44-patient trial done by Curtis et al. has demonstrated beneficial effects of the cannabinoid-treatment on aspects such as chorea, behavior, cognition, and motor and neuropsychiatric symptoms [144].

By administering AM404 and UCM707 (ECB re-uptake inhibitors), a reduction of hyperkinetic activity and restoration of neurochemical alterations in an HD rat model with bilateral striatal injection of 3-nitropropionic acid (3-NP) was observed. In the case of AM404, the activation of TRPV1 receptor was responsible for the effect, without the involvement of CB1R [1,145]. 3-NP has a damaging effect on the striatum due to its inhibition of a component of the respiratory chain [146].

CP55,940, which is a CB1R agonist, has demonstrated beneficial anti-hyperkinetic effects with no influence on GABA and dopamine levels [1]. Chronic administration of WIN 55,212-2 (another CB1R agonist) in a R6/1 transgenic mouse model of the disease demonstrated a protective effect against motor impairment. Concerning nabilone, studies have shown that its administration did not have a beneficial effect on the hyperkinetic component associated with the disease. This could be due to the fact that it does not determine the activation of TRPV1 receptors, which are responsible for the anti-hyperkinetic activity in the rat model of the disease. It is important to note the involvement and beneficial aspects of using compounds that can activate both CB1 and TRPV1 receptors [147].

In regard to the neurodegenerative aspects of HD, studies realized on rodent models of the disease have demonstrated the beneficial effect of compounds that act as CB1R agonists [54]. In a rat model of HD in which excitotoxicity was increased through striatal injection of quinolinic acid, by administering WIN 55,212-2, a reduction of both glutamate levels, as well as quinolinic acid effect on corticostriatal local field potential recordings, was observed in vitro. The observed-effects were CB1-dependent, which was demonstrated by the administration of AM 251 (CB1R antagonist), with this compound blocking the effects of WIN 55,212-2 [148].

Δ^9^-THC determined an improvement of symptoms, neuropathology and molecular pathology related to R6/2 model of HD [136]. These findings support the role of CB1R and CB1 agonists as useful options for reducing or delaying the progression of HD.

The administration of HU-308 (CB2R agonist) has shown promising neuroprotective effects in quinolinic-acid lesioned mice as well as in the malonate-lesion rat model of HD through the reduction of neuronal damage in the striatum, by attenuating glial activation [140]. R6/2 mice, which are deficient in CB2R, were associated with a faster progression of the disease phenotype, with the ablation leading to an increase in the activation of the glia as well as to a higher sensitivity to excitotoxic-induced neurodegeneration of the striatum [140].

Another way through which some cannabinoids can protect against neurodegeneration is independent of CB1R and CB2R and involves ROS blockage (e.g. Δ^9^-THC) [149].

In a study realized by Heim et al., the use of nabilone was assessed in five patients with HD for alleviating therapy-resistant symptoms. All patients reported improvement of symptoms, suggesting that nabilone is well tolerated and could be an efficient adjuvant in the treatment of HD [150].

In a literature review done by Akinyemi et al., it has been found that for the selected human studies, medical marijuana demonstrated therapeutic effects for movement disorder symptomatology. These studies used nabilone (synthetic cannabinoid capsule that is a selective agonist for CB1R and CB2R), and Sativex®, which is an oral spray that contains a combination of Δ^9^-THC and CBD. The majority of studies demonstrated statistically significant results favoring the use of medical marijuana for improving quality of sleep and motor symptoms. Positive effects have also been shown for symptoms such as spasticity and tremor, although patients that presented these symptoms had other neurodegenerative diseases and not HD (e.g., spasticity improvement was demonstrated in patients with autoimmune demyelinative disease and MS, while tremor was improved in patients with PD following administration of medical marijuana). The positive effects shown in patients with HD were improvements in chorea; improvements in the neuropsychiatric index; and trend for improvements in the Unified Huntington’s Disease Rating Scale motor score, the dystonia subscore and behavior score [151].

In another report done by Saft et al., 7 patients with early-onset HD, in which dystonia was the main manifestation, were treated with cannabinoids. Five of these patients were in advanced disease stages. It was shown that the treatment demonstrated beneficial effects on dystonia, thus improving motor symptoms. Improvements that were reported by participants include nail and dental care, social-life activities, fine motor skills and movements such as head-lifting, thus leading to better life-quality. Three of these patients have also reported behavioral changes, with less irritability and apathy [152].

Another compound of interest is cannabigerol (CBG), which shares characteristics with CBD and Δ^9^-THC regarding affinity and activity but also presents uniqueness in that it interacts with α-2 adrenoreceptors and with 5-hydroxytryptamine (5-HT_1A_). Research, which used this compound as well as VCE-003.2 (a synthetic quinone derivative), demonstrated in vitro neuroprotective potential in disease-models such as HD, ALS, PD and MS, reducing the severity of neurologic illnesses. In a 3-NP animal model of HD, it has shown that the use of CBG prevented neuronal death within the striatum, also improving motor deficits and reducing inflammatory markers [153].

Another study that used VCE-003.2 discovered the beneficial effects of the compound on the symptoms of the disease in the quinolinic acid murine model. The animals showed better Rotarod performance. In the 3-NP model of HD, VCE-003.2 demonstrated good results for the improvement of motor deficits [154].

The specific cannabinoid tetrad (hypothermia, hypomotility, antinociception and catalepsy) is not induced by CBD; therefore, the compound could be of use for the management of movement disorders, aspect that is also highlighted by a series of pre-clinical and clinical studies. In a cell culture model of HD in which cells expressed mHTT, CBD along with other tested compounds (Δ^8^-THC, Δ^9^-THC and CBD) have shown a protective effect against cell-death induced by HTT [155], although these effects might be independent of CB1 and due to antioxidant mechanisms. CBD has also exerted protective effects in 3-NP animal models of the disease by reversing or attenuating the alterations that were induced by 3-nitropropionic acid [146]. Recent data from literature regarding the involvement of ECS in various pathological aspects of HD is presented in Table 3.

## 6. Cannabinoids and Multiple Sclerosis

MS is a neuroinflammatory condition, characterized by chronic disability of the CNS, being spread in young adults aged between 20 and 40 years [156]. Recent prevalence studies reveal that an estimated population of 2.8 million people worldwide is suffering from MS [157,158,159]. The prevalence of the disease is reported to be of 35.9 cases per 100,000 people and has been constantly growing in recent decades; it was observed that women are more affected than men by the disease, by a ratio of up to 3:1 [160].

It causes significant multifocal demyelination in both gray and white matter, and axonal damage via an autoimmune mechanism directly linked to neuroinflammation [161]. The stimulation of the immune system is not completely understood [162], but it is believed that both genetic and environmental factors are the main triggers [12].

Both adaptive and innate immunity are involved in the inflammatory process [163,164]. Myelin-specific CD4 and CD8 positive T-lymphocytes of adaptive immunity have been identified within active MS lesions. B-cells play a key role in sustained inflammation of progressive MS by stimulation of T cells response [165]. The negative influence on myelin sheaths through their infiltration into CNS by crossing BBB is due to release of pro-inflammatory cytokines during macrophage activation, including TNF-α, lymphotoxin, interleukin-6 and metalloproteinases [49,166]. Recent studies indicate a risen presence of ectopic B-cell follicles in the cerebral meninges that is associated with irreversible disability and cortical demyelination [165,167].

Oligodendrocyte degeneration in the CNS is another important mechanism involved in disease pathology [168]. Recent research shows that oligodendrocytes, also known as myelin-forming cells located in CNS, play a key role in remyelination process that occurs in early phases of the disease that temporarily decreases the symptoms in relapsing-remitting MS [169,170]. Microglia, the innate immune cells in the CNS and activation of astrocytes, a sub-type of glial cells in CNS, represent early events in lesion development, loss of blood–brain barrier function and CNS inflammation [171]. A release of proteolytic enzymes, cytokines, reactive oxygen and nitrogen species is observed with negative influence on oligodendrocytes and myelin [172].

The condition is characterized by episodic relapses of different intensity and remissions and leads to progressive reduction of neurological function due to disruption of neural transmission [12]. The consequences are represented by prolonged and progressive physical, psychological and cognitive impairments [173,174]. The signs and clinical symptoms of MS vary depending on the CNS damage and include paresthesia, dysesthesia, weakness and visual disturbances [175]. Spasticity is the mainly observed symptom in MS. It is associated with spasms, pain and sleep disturbance [176,177]. Another symptom frequently reported by up to 80% of patients suffering from MS is urinary incontinence due to bladder dysfunction [178].

According to International Advisory Committee on Clinical Trials of MS in 2013, based on the classification made by Lublin et al., four main clinical courses of the disease have been identified, depending on its evolution: relapsing-remitting type (RRMS), progressive relapsing type (PRMS), primary-progressive type (PPMS) and secondary-progressive type (SPMS) [179,180]. RRMS is reported to have the highest prevalence, being encountered in approximately 85% of cases [179]. The PPMS and SPMS pathophysiology is different from that of RRMS [18].

Even though is known that MS has no cure and drugs used for progressive forms are currently limited, the main therapeutic goal remains the reduction of the exacerbations of inflammatory phenomena and neurological disability of the patients [181]. This is achieved by modulation of innate and adaptive immune responses with disease modifying therapies (DMTs) in long-term treatment [182]. Being a chronic disease, it is very important to optimize MS pharmacotherapy in order to obtain the best clinical response from the patient [183]. Using immunomodulating or immunosuppressive drugs is unfortunately accompanied by high risk of cancer development due to involvement of immune system in recognizing and eliminating cancer cells [182]. On the other hand, some available MS DMTs have been used for years in cancer therapy (e.g., rituximab [184], cladribine [185] and methotrexate [186]), while other current DMTs are being evaluated in the present for their anti-tumor potential (e.g., dimethylfumarate [187], fingolimod [188] and teriflunomide [189]).

Cannabinoids have positive effects in reducing MS symptoms and can limit the inflammatory processes ongoing in the CNS and progressive neurodegeneration in MS, due to anti-inflammatory properties and neuroprotective effects [12,190]. While activation of CB2R demonstrates the modulation of inflammatory process; through inhibition of neuroinflammatory signaling pathways, CB1 agonists are involved in neuroprotection [191]. Both exogenous or endogenous cannabinoids also influence humoral and cellular immune response [50].

A combination of equal amounts of Δ^9^-THC and CBD was recently approved in some countries under brand name Nabiximol (Sativex^®^) [192], which is used for the management of bladder dysfunction, neuropathic pain and spasticity associated with MS [193]. Wade et al. reported significant improvement in spasticity after administration of a daily dose below 120 mg of Sativex [194]. Surprisingly, some researchers reported that MS-induced spasticity was controlled only by Δ^9^-THC action on CB1Rs and not by CBD [195]. Despite the fact that CBD has low affinity to both CB1R and CB2R, recent studies showed the ability to potentiate the effect of Δ^9^-THC due to its allosteric modulation property and CBR indirect antagonism [196]. Another randomized double-blind placebo-controlled study reported that the treatment with a daily dose consisting of 48 sprays of Sativex^®^ during 5 weeks reduced MS-related pain [197]. The patients enrolled in this study were tracked for the next 2 years, and it was observed that the effect was maintained, with no signs of tolerance [198].

Preclinical studies on different experimental animal models of MS show a wide alteration of CB1R and CB2R expression [199]. Phytocannabinoids promote reduction of inflammation and neuroprotection [19]. Their anti-inflammatory and antioxidant properties mediate the neuroprotection towards modulation of signaling systems, receptors or channels involved in neurodegenerative diseases [200]. It was also reported that agonists of both ECB receptors can limit relapses, axonal degeneration and neuroinflammation in rodent models of MS [192].

It is of interest to elucidate the mechanism through which they influence neuroinflammation and neurodegeneration since this could contribute to the development of new therapeutic approaches.

Experimental autoimmune encephalomyelitis (EAE) is frequently used as rodent model of autoimmune disease of the CNS that is characterized by brain inflammation associated with neurodegenerative pathology, including the development of cutaneous mechanical and cold hypersensitivity. It initiates demyelination and neurological dysfunction within acute monophasic RRMS and SPMS [201]. Within this model, animals are immunized with myelin antigens such as proteolipid and myelin basic proteins; encephalitogenic peptides; or myelin oligodendrocyte glycoprotein (MOG). Another possibility of inducing EAE is by T lymphocyte sensitization to myelin proteins due to adoptive transfer of encephalitogenic T cells [202]. The immune response consists of demyelination process and progression of CNS inflammation, involving paralysis of animal tail, limb and forelimb.

The influence of cannabinoids on EAE-induced neurodegeneration phenomenon is due to their affinity on both CB1R and CB2R.

In a report done by Baker et al., mice suffering from chronic relapsing EAE were treated with Δ^9^-THC, methanandamide (analogue of AEA), WIN 55,212-2 (agonist of both CB1R and CB2R) and JWH-133 (agonist of CBR type 2). The treatment reduced motor symptoms, including limb spasticity, tremor and paralysis [203]. It was hypothesized that it could control clinical EAE symptoms through inhibition of synaptic transmission [204].

Similar studies were conducted by different research teams identifying the importance of these receptors in controlling neuroinflammation [205,206,207,208,209,210,211]. Ni et al. showed that administration of WIN 55,212-2 in C57BL/6 mice immunized with MOG_35–55_ attenuated EAE progression [209]. In addition, Maresz et al. revealed the effects of co-administration of CB1R/CB2R antagonists and WIN 55,212-2 on EAE progression. Thus, administration of CB1R antagonist SR141716A had no influence on the protective effect, whereas the CB2R antagonist SR144528 reduced it, suggesting that CB2Rs play a key role in the protective effect of WIN 55,212-2 [212]. Their activation produces inhibitory effects on leukocyte/endothelial interactions that have beneficial effects in disease management [209]. On the other hand, CB1R activation may provide neuroprotective effects in later stages of the disease, although CB1R blockers did not reduce the beneficial effects of WIN 55,212-2 [212]. These aspects suggest that only neuronal but not T lymphocyte CB1 expression plays an important role in mediating EAE suppression [213].

It was also observed that during chronic relapsing, EAE mice deficient in CB1Rsare are more susceptible to neurodegeneration following immune attack. In this process, both markers of axonal damage such as caspase 3 activation and presence of nonphosphorylated form of neurofilament H epitope are involved [49]. In another experimental model using Lewis rats, it was observed that EAE induction reduced CB1R activation in striatal and cortical neurons [214,215].

Neuroprotective CB1R-mediated effects are explained due to modulation of glutamate activity, control of NMDA receptor-induced calcium influx and decrease in oxidative injury by antioxidant properties [67]. Increased glutamate level is associated with EAE neurodegeneration due to its excitotoxicity [210,216]. Several studies reported that Δ^9^-THC and its synthetic analogues demonstrated antiglutamatergic effects by inhibiting the release of glutamate in rat hippocampus cultures [217].

The inflammatory response in EAE model is mediated by CB2Rs expressed by infiltrating T-cells and monocytes [218]. CB2Rs are also found in microglial cells; thus, their activation inhibits the production of proinflammatory cytokines and oxygen and nitrogen reactive species. It was observed elevated local levels of interleukin-6 (IL-6) in the CNS of animals with induced EAE, although it was not present in their spleens [219].

Kozela et al. administrated 5 mg/kg b.w. CBD (i.p.) in C57BL/6 mice for 3 consecutive days. The dose that was used in the experiment is similar to the one used in studies on rheumatic arthritis. The histological analysis showed a reduction of the severity of EAE through different mechanisms: diminishment of axonal damage and inflammation, decrease of spinal glial activation and inhibition of T-cell recruitment in the spinal cord [220]. Gonzáles-García et al. performed a similar study and observed that different negative aspects of the disease such as microglial activity, cell infiltration and demyelination, axonal damage and levels of pro-inflammatory cytokine IL-6 were significantly reduced after administration of a dose of 50 mg/kg b.w. CBD [221]. Similarly, Giacoppo et al. revealed the neuroprotective effect of CBD on apoptotic markers associated with neurodegeneration in MS [222].

Theiler’s murine encephalomyelitis virus (TMEV) belongs to *Picornaviridae* family, being a nonenveloped RNA virus. This model has been used as viral model for MS [223]. TMEV infection induces inflammatory demyelination process attributed to infiltration of both CD4 and CD8 positive T cells into CNS. Whilst CD4 positive T-cells are important in development of demyelinating disease at later stages, CD8 positive T-cells make a significant contribution in early phase of immune response against the virus [224]. It is supposed that autoimmune response in propagating the disease is due to myelin antigens/epitope spreading [225]. On the other hand, it was also observed that depletion of CD8+ T-cells reduced chronic disease at the same time, with chronic axonal injury or neurodegeneration [223].

Different data indicate that cannabinoids can reduce MS progression induced by this murine model. In this respect, Molina-Holgado et al. showed that ECB AEA was found to potentiate IL-6 (immunosuppressive and anti-inflammatory cytokine) production by TMEV-infected astrocytes in a dose-dependent manner [219].

Other researchers demonstrated the anti-inflammatory role of AEA in MS due to inhibition of IL-1β, IL-6, IL-12 and IL-23 release in myeloid dendritic cells and inhibition of microglial activation [161]. IL-12 leads to induction of Th1 regulatory cells, while IL-23 is a maintenance factor of Th17 phenotype [226].

The neuroprotective effect of AEA against microglial neurotoxicity was mediated by a mechanism between neurons (with reference to membrane glycoproteins) and microglia/macrophages that involves CD200 ligand-receptor interaction [227].

Another study done by Loria et al. described that treatment with palmithoylethanolamide (PEA), an analogue of AEA with anti-inflammatory activity, reduced the expression of IL-1, TNF-α and microglial activation in the spinal cord of mice with TMEV-induced demyelinating disease [228].

Significant improvement in motor function and neurological deficits was obtained following the administration of UCM707, a selective AEA uptake inhibitor in the same animal model or different synthetic cannabinoids such as JWH-015, WIN 55,212-2 and ACEA. The proposed common mechanisms through which the compounds influence disease progression were the reduction of microglial activation, inhibition of major histocompatibility complex (MHC) class II antigen expression, depletion of spinal-cord-infiltrating CD4 positive T cells or decrease of the production of pro-inflammatory (TNF-α, IL-1β and IL-6) cytokines [229,230].

Cannabinoids produce immunosuppresion in astrocyte reactivity. During MS progression, astrocytes are activated and contribute to inflammatory response by cytokines, chemokines and nitric oxide release.

It was observed that AEA inhibited production of IL-6 and astrocytes activation through CB1-mediated pathway, in a study on primary astrocytes infected with TMEV [231].

By using different mouse models of MS, the above-mentioned findings suggest that both endogenous and synthetic CBR ligands can influence the development of progressive forms of MS.

In the following, a summary of recent literature data regarding the implications of ECS in various pathological aspects of MS is presented in Table 4.

## 7. Conclusions

Neurodegenerative diseases affect millions of people worldwide. Whilst there are currently no available cures, the management of symptoms and improvements of physiological and cognitive deficits remain a clinical challenge. The ECS is an attractive research field involved in different types of interactions with neurotransmitters involved in the pathological aspects of neurological diseases. The complexity of ECB functions offers new insights into the mechanisms that underlie the neuroinflammatory and neurodegenerative components of diseases such as AD, PD, HD and MS. The current review provides information regarding the CBR and their endogenous or exogenous ligands, contributing to a better understanding of pathophysiological features as well as allowing the identification of promising and effective therapeutic candidates for the treatment of these disorders. Despite the advantages of the reviewed compounds, further preclinical and clinical studies that evaluate safety, efficacy and pharmacokinetics are necessary.

## Figures and Tables

**Table 1:** Involvement of ECS in various pathological aspects of AD.

Mechanisms of AD Pathogenesis	Implications of the ECS in AD		Cannabinoid Receptor Ligands with Potential Benefits in Therapeutic Management of AD		
	Target Components	Physiological Function	Disease Model and Species	Compound	BIOLOGICAL EFFECT
β-amyloid (Aβ) peptides →neurodegenerative cascade → neuronal cell death [35,75,79,80]	CB1R activation 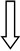	hyperpolarization of the neuronal membrane modulating of neurotransmitter and cytokine release [79]	Rats intra-CA1 microinjection i.p. intra-NAc intra-mPFC	ACPA (agonist of CB1R)	cognitive impairments [52,81]
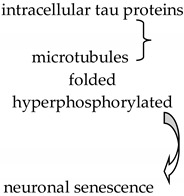 [35,75,79,80]	↑ CB1R density	neuroprotective and anti-inflammatory response [79]	Rats intra-CA1 microinjection intra-NAc intra-BLA	AM251 (antagonist of CB1R)	enhanced memory and learning processes [52,81]
			Rat hippocampus	CBD and Δ^9^-THC	↑ synthesis of tryptophan → improvement of the disease [82]
			In vitro Methods Molecular docking	Cannabinoids (CBD, CBD-DMH)	↓ of oxidative stress ↓ of TNF-α synergistic effect with AchE inhibitors → preventing Aβ formation improvement of circadian rhythm disturbances commonly seen in AD [79,83]
proteolytic cleavage of β-APP → formation of Aβ1-42 monomers → activation of astrocytes and microglia → release of inflammatory cytokines, kinases and nitric oxide → phosphorylation of tau proteins [84]	Endocannabinoids (2-AG) CB1R/CB2R activation	synthesized by microglia and astrocytes → suppress cytokine synthesis via CB1/CB2 receptors	Primary hippocampal neuron cell cultures from rat embryos	MAGL inhibitors (URB 602 andJZL 184)	↑ endogenous levels of 2-AG → ↓ of TUNEL-positive neurons anti-apoptotic effects mediated through CB1R [85]
(added space) GSK-3β promotes tau proteinhyperphosphorylation → formation of NFT → impairing the axonal transport → neuronal atrophy [75]		neuroprotective effects → targeting this pathway with key roles in AD pathogenesis	PC12 cells treated with Aβ	CBD AEA WIN 55,212–2	attenuation of tau hyperphosphorylation by inhibiting Gsk-3β [75]
increased COX-2 levels → involvement in neuroinflammation [86]		2-AG is substrate for COX-2 2-AG suppress elevation of hippocampal COX-2 expression		MAGL inhibitor (URB602) and nonselective MAGL inhibitor (ATFMK)	↑ 2-AG levels → suppresses the expression of COX-2 action mediated by CB1R [86]
tau pathology [87]	CB2R activation	role in memory processing its activation → vital for cognitive processes a depletion or disruption of these receptors in rodents →induces long-lasting memory deficits	Rats	AM630 (CB2R antagonist)	negative effects such as impaired memory [87]
			Rats → impaired memory by administration of okadaic acid	JWH-133 (CB2R agonist)	reduced spatial memory impairment reduced neuroinflammation and neurodegeneration [87]
formation of Aβ peptide aggregates in the brain [88] PPAR- γ involvment in disease management [89]	CB2R activation	involved in controlling Inflammation	Beta-amyloid challenged astrocytes	CBD ± PPAR- γ antagonist (MK886 or GW9662)	interacts with the PPAR-γ receptor → attenuate beta-amyloid (Aβ)-induced neuroinflammation promote neurogenesis in the hippocampus [89]
Aβ peptide-induced neurotoxicity, oxidative stress and inflammatory status	CB2R activation	enhances immune system response andautophagy pathway	Analyses of transcriptome of APP/PS1 mice hypocampus	CBD, chronic i.p. injection (30 days)	improvement of the neuroinflammation and oxidative stress level [90]
neuroinflammatory mechanism	CB2R activation	ameliorate the neuroinflammation and cognitive impairments of AD	APP/PS1 mice	JWH015	improvement of novel object recognition regulation in microglia-mediated neuroinflammation [92]
evidence that associates neutrophil-derived myeloperoxidase (MPO) in the pathogenesis of AD	CB1R activation		Murine model (male mice) induced with focal cerebral ischaemia	Δ^9^-THC and SR141716 (CB1-R antagonist)/ AM630 (CB2R antagonist)	CB1R antagonist inhibited the neuroprotective effect of Δ^9^-THC CB2R antagonist had no effect reduction of the size of cerebral infarction given by MCA affective, cognitive, sensory and somatic effects, neuroprotective properties [52,75]
		mechanism of inhibition of myeloperoxidase independent of the cannabinoid receptor		CBD	(added space) the neuroprotective effect CBD was not inhibited by both CB1R and CB2R antagonist → effects, independent of cannabinoid receptors inhibition of MPO activity in neutrophils →anti-inflammatory mechanism pre- and post-ischaemic neuroprotective effect [91]
β-amyloid (Aβ) plaques cause injuries in the pulvinar nucleus → disruption of thalamo-cortical circuits including disturbances in visual attention [93]	CB1R NAPE-PLD FAAH located in the thalamus - pulvinar nucleus (lateral, medial and inferior) - dorsal lateral geniculate nucleus	physiological connections withprefrontal cortex and amygdala [94,95,96,97]	Coronal brain sections from Vervet monkey		lesioning of pulvinar nucleus → disturbance in perception of distracting stimuli [98] modulation of visual and spatial perception and processing mechanisms plasticity phenomena involving subcortical visual pathways synchronization of neural activity [99] (added space)
			Patients with AD	Δ^9^-THC CBD	↓of the lateral pulvinar nucleus ofAD patients [98] regular consumtioninterferes with emotional face recognition [100]

Legend: CA1: dorsal hippocampus; BLA: basolateral amygdala; NAc: nucleus accumbens; PFC: prefrontal cortex; and CBD-DMH: cannabidiol dimethylheptyl; ↓: decrease; ↑ = increase.

**Table 2:** Implications of ECS in various pathological aspects of PD.

Mechanisms of PD Pathogenesis	Implications of the ECS in PD		Cannabinoid Receptor Ligands with Potential Benefits in Therapeutic Management of PD		
	Target Components	Physiological Function	Disease Model and Species	Compound	Biological Effect
↓ tyrosine-hydroxylase-positive neurons in the substantia nigra pars compacta down-regulation of CB2Rs in the substantia nigra	CB2R	neuroprotective effect are involved in neuroinflammation [25,105]	MPTP-induced mouse model of Parkinson’s disease	WIN 55,212 –2JW015 (CB2 receptor agonist)	protects neuron loss reduces MPTP-induced microglial activation reverses MPTP-associated motor deficits [37] reduce inflammation in the brain of MPTP-treated mice [25]
side effects of current anti-parkinsonian therapies, especially L(3,4) dihydroxyphenylalamine L-DOPA-induced dyskinesia [108]	CB1R	modulation of neurotransmission and contribution to synaptic plasticity [108]		WIN 55,212–2 HU210	protected nigrostriatal dopamine neurons reduced microglia activation [109]
↓ dopaminergic neurons in the substantia nigra compacta and a significant reduction of striatal dopamine [104]	CB2R	CB2R-deficient mice showed an exacerbation of PD pathology [25]		AM1241 (selective CB2R agonist)	regenerated dopaminergic neurons reversed the decreased CB2R level in the PD mouse brain [104]
currently therapy for PD is symptomatic whose efficacy is limited due to side effects	CB1R	neuroprotective properties against excitotoxicity and oxidative stress neuroinflammation, which are also associated with PD [104]	Clinical study on PD patients	CBD	change in patients’ lives possible neuroprotective effects assessed [106]

**Table 3:** Involvement of ECS in various pathological aspects of HD.

Mechanisms of HD Pathogenesis	Implications of the ECS in HD		Cannabinoid Receptor Ligands with Potential Benefits in Therapeutic Management of HD		
	Target Components	Physiological Function	Disease Model and Species	Compound/Intervention	Biological Effect
mHTT - direct repressive effect on CB1R gene transcription→ Loss of CB1R binding in the striatum→ Reduction of CB1R [130,133]	CB1 Ractivation	CB1R are necessary to counteract neuronal degeneration [133] Activation of CB1R pathway is associated with a protective effect [135] CB1R activation protects neurons from NMDA-induced excitotoxicity and inhibits presynaptic release of glutamate [60]	R6/1 transgenic HD mouse model	WIN 55,212-2	Antihyperkinetic activity prevention of motor impairment [147]
				Environment enrichment	Upregulation of CB1R binding → behavioral improvement [134]
			R6/2 mouse model of HD	Δ^9^-THC	↓ of motor coordination deficits improvement of motor and exploratory behavior ↓ of striatal atrophy and HTT aggregate accumulation [136]
			3NP animal model of HD	CBD	Reversibility or attenuation of alterations induced by 3NP [146]
				CBG	Prevention of striatal neuron death Improvement of motor deficits Reduction of inflammatory markers [153]
			R6/2 model of HD	GAT211 GAT228 GAT229 (positive allosteric modulators)	Improvement measures of health GAT211 and GAT229 reduced psychoactivity, without tolerance or dependence [137,138,139]
			N171-82Q transgenic model	CB1R gene inactivation	Earlier and exacerbated motor alternations Increased striatal aggregation frequency [136]
			3NP animal model of HD		CB1R are necessary to counteract neuronal degeneration [133]
			Rat model of HDExcitotoxicity was increased through striatal injection of quinolinic acid	WIN 55,212-2 CBD	Decreased bothglutamate levels and the effect of quinolinic acid on corticostriatal local field potential recordings [148]
			Cell culture model of HD with mHTT expressive cells	CBD Δ^8^-THC Δ^9^-THC	51–84% protection against HTT-induced cell death [155] Remark: Effects might be independent of CB1R and due to antioxidant mechanisms
Microglial CB2R → induced in HD patients and animal models CB2R ablation exacerbates microglial activation and accelerates appearance of symptoms [140]	CB2R	CB2R activation → neuroprotective effect in HD models → control of deleterious microglial activity [140]	Quinolinic-acid lesioned mice model of HD	HU-308	Reduction of neuronal damage in the striatum by attenuating glial activation [140]
			Malonate-lesion rat model of HD		Accelerated progression of the HD phenotype Increased glial activation Higher sensitivity to striatal neurodegeneration induced by excitotoxic processes [140]
			R6/2 mice model	CB2R ablation	Faster progression of the disease phenotype Increased glial activation Higher sensitivity to striatal neurodegeneration induced by excitotoxic processes [140]
	CB1R/CB2R		Human studies-patients with HD	Nabilone Sativex^®^	Improvements in chorea Improvements in the neuropsychiatric index Trend for improvements in the Unified HD Rating scale motor score, dystonia subscore and behavior score [151]
			Human studies-patients with early-onset HD	Nabilone Sativex^®^ Dronabinol	Improvement of dystonia Quality of life improvement Behavior improvement [152]
	TRPV1		HD rat model with bilateral striatal injection of 3NP	AM404 (ECB reuptake inhibitor)	Reduction of hyperkinetic activity and restoration of neurochemical alterations [1,145]

**Table 4:** Implications of ECS in various pathological aspects of MS; ↓: decrease; ↑ = increase.

Mechanisms of MS Pathogenesis	The Endocannabinoid System and Its Implications in MS		Cannabinoid Receptor Ligands with Potential Benefits in Therapeutic Management of MS		
	Target Components	Physiological Function	Disease Model and Species	Compound	Biological Effect
spasticity → the mainly observed symptom in MS is associated with spasms, pain and sleep disturbance [176,177]	CB1R and CB2R	CB1R inhibits synaptic transmission → main target for control of spasticity [204]	chronic relapsing EAE	Δ^9^-THC methanandamide (analogue of AEA) (CB1R agonists) WIN 55,212-2 (CB1R/CB2R agonist) JWH-133 (CB2-R agonist)	amelioration of some motor symptoms such as limb spasticity, tremor and paralysis [203]
inflamamation→ recruitment of leukocytes from the blood into the CNS adhesion to endothelial cells (added space) cerebrospinal fluid: increased glutamate level, differential expression of glutamate receptors [232] increased glutamate level → neurodegeneration due to excitotoxicity [210,216]	CB1R and CB2R	CB2R have immunomodulatory properties [209]	EAE induced C57BL/6 mice immunized with MOG_35–55_ + pertussis toxin	WIN 55,212-2 SR 141716A (CB1R antagonist) SR144528 (CB2R antagonist)	CB1R antagonist → no influence on the protective effect 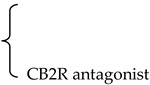 → key role in the protective effect of WIN55212-2 [212] 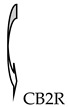 stimulation → attenuated EAE progression potential target to inhibit leukocyte trafficking in EAE [209]
	CB1R	activation of cannabinoid receptors inhibits the release of glutamate presynaptically [217]	Rat hippocampal neurons culture	AEA Memantine Δ^9^-THC	(added space) antiglutamatergic effects by ↓ of Mg^2+^ concentration →↓ excitation level in the entire network of neurons in the culture glutamatergic excitatory postsynaptic currents elicited by direct stimulation of the presynaptic neuron [217]
			EAE induced C57BL/6 mice—i.p. administration for 3 consecutive days	CBD	(added space) Low dose of CBD → ↓ inflammation → axonal damage ↓ spinal activation of glia inhibition of T-cell migration in the spinal cord [220] High dose of CBD → ↓microglial activity↓ ↓cell infiltration and demyelination ↓axonal damage ↓levels of IL-6 [221]
	CB1R and CB2R	involvement in treating of neurodegenerative diseases driven by chronic neuro-inflammation	EAE-induced C57BL/6 mice immunized with MOG_35–55_ + pertussis toxin	Δ^9^-THC + CBD	Δ^9^-THC + CBD→attenuates the development of EAE [157]
	CB1R	immunosuppressive effects on astrocytes	In vitro method TMEV-infected astrocytes	AEA	dose-dependent potentiating of IL-6 [219] inhibition of astrocytes activation →the production of IL-6 [231] inhibition IL-1β, IL-6, IL-12 and IL-23 release in myeloid dendritic cells inhibition of microglial activation [161]
			Mouse model TMEV-induced demyelinating disease	PEA	↓ expression of IL-1, TNF-α↓ microglial activation in the spinal cord of mice [228]
				UCM707 WIN 55,212-2 JWH-015 ACEA	↓ microglial activation inhibition of MHC class II antigen expression ↓ of spinal cord infiltrating CD4T cells– ↓ the production of IL-1β, IL-6 and TNF-α [229,230]

## Data Availability

No new data were created or analyzed in this study. Data sharing is not applicable to this article.

## Abbreviations

2-AG	2-Arachidonoylglycerol
3-NP	3-Nitropropionic acid
5-HT1A	5-Hydroxytryptamine
AchE	Acetylcholinesterase
ACPA	Arachidonyl-cyclopropyl amide
AD	Alzheimer’s Disease
AEA	Anandamide
ALS	Amyotrophic lateral sclerosis
APP	Amyloid precursor protein
BBB	Blood–Brain Barrier
BLA	Basolateral amygdala
CA1	Dorsal hippocampus
CB1R	Cannabinoid-receptors type 1
CB2R	Cannabinoid-receptors type 2
CBD	Cannabidiol
CBD-DMH	Cannabidiol dimethylheptyl
CBG	Cannabigerol
CBR	Cannabinoid receptor
CIDP	Chronic inflammatory demyelinating polyneuropathy
CNS	Central nervous system
COX	Cyclooxygenase
DMT	Disease modifying therapy
EAE	Experimental autoimmune encephalomyelitis
ECB	Endocannabinoid
ECS	Endocannabinoid system
FAAH	Fatty acid amide hydrolase
GABA	Gamma-aminobutyric acid
GBS	Guillain–Barré syndrome
GPRC	G-Coupled protein receptor
GSK-3β	Glycogen synthase kinase-3β
HD	Huntington’s Disease
HTT	Huntingtin
IL	Interleukin
i.p.	Intraperitoneal
L-DOPA	Levodopa
MAGL	Monoacylglycerol lipase
MAPK	Mitogen-activated protein kinases
MCA	Middle cerebral artery
MHC	Major histocompatibility complex
mHTT	Mutant huntingtin
MOG	Myelin oligodendrocyte glycoprotein
MPTP	1-Methyl-4-phenyl-1,2,3,6-tetrahydropyridine
MS	Multiple Sclerosis
MSN	Medium spiny neurons
NAc	Nucleus accumbens
NAPE-PLD	N-arachidonoyl phosphatidylethanolamine-specific phospholipase D
NF-kB	Nuclear factor-kB
NMDA	N-Methyl-D-aspartic acid
NTF	Neurofibrillary tangles
OKA	Okadaic acid
PD	Parkinson’s Disease
PEA	Palmithoylethanolamide
PFC	Prefrontal cortex
PG	Prostaglandin
PKA	Protein kinase A
PPMS	Primary-progressive MS
PRMS	Progressive-relapsing MS
ROS	Reactive oxygen species
RRMS	Relapsing-remitting MS
SPMS	Secondary-progressive MS
TMEV	Theiler’s murine encephalomyelitis virus
TNF-α	Tumor necrosis factor-α
TRPV	Transient receptor potential vanilloid
VTA	Ventral tegmental area
Δ^9^-THC	Δ^9^-Tetrahydrocannabinol
